# Autologous T-cell therapy based on a lentiviral vector expressing long antisense RNA targeted against HIV-1 *env* gene influences HIV replication and evolution *in vivo*

**DOI:** 10.1186/1742-4690-10-S1-O46

**Published:** 2013-09-19

**Authors:** Alexander Pasternak, Nikolay Korokhov, Ben Berkhout, Vladimir Lukashov, Laurent Humeau

**Affiliations:** 1 Academic Medical Center of the University of Amsterdam, Department of Medical Microbiology, Amsterdam, The Netherlands; 2 VIRxSYS Corporation, Gaithersburg, USA

## Background

We report the results of a Phase II clinical trial of VRX496, an HIV-based vector encoding a 937-nt long antisense (AS) RNA targeting the gp120 coding region of HIV-1 *env* gene [1-3].

## Materials and methods

Autologous CD4^+^ T lymphocytes from HIV-infected subjects were genetically modified *ex vivo* with the vector, expanded, and 10-80 billion vector-modified cells were reinfused into patients. Longitudinal effects of the therapy on HIV-1 *env* evolution were analyzed in 17 subjects sampled both pre-infusion and monthly post-infusion for 6 to 12 months. Plasma-derived viral RNA from 144 samples was amplified, cloned, and the full-length gp120 coding region was sequenced in 8-10 clones for each sample.

## Results

Two AS-related factors: (a) sequence similarity of the AS RNA with the targeted HIV transcripts at baseline, and (b) persistence of the infused vector-modified cells during the follow-up period, independently and cooperatively influenced the following parameters: (i) pairwise genetic distances of the gp120 conserved region sequences in the post-infusion samples to the corresponding pre-infusion samples, (ii) change in viral diversity from baseline, (iii) genetic distances of the post-infusion viral quasispecies to the most recent common ancestral sequence (MRCA) of all patient’s viral sequences, relative to the MRCA distances of pre-infusion viruses, and (iv) change of HIV-1 plasma RNA load from baseline (*P* < 0.005 for all parameters). The effects of AS vector on virus evolution were stronger for the AS-targeted region of gp120 than for the untargeted region. The degree of virus evolution from the pre-infusion to the post-infusion quasispecies (relative MRCA distances) negatively correlated with virus replicative fitness, assessed *ex vivo* by growth competition assay.

## Conclusions

The same AS-related factors were associated with accelerated virus evolution and with the relative decrease in plasma viral load, suggesting that selective pressure exerted by the AS causes directional virus evolution, presumably towards escape, which is associated with the fitness loss.
